# Spatial distribution and determinants of undernutrition among reproductive age women of Ethiopia: A multilevel analysis

**DOI:** 10.1371/journal.pone.0257664

**Published:** 2021-09-20

**Authors:** Ayenew Kassie Tesema, Alemneh Mekuriaw Liyew, Adugnaw Zeleke Alem, Yigizie Yeshaw, Getayeneh Antehunegn Tesema, Achamyeleh Birhanu Teshale

**Affiliations:** 1 Department of Health Education and Behavioral Science, Institute of Public Health, College of Medicine and Health Sciences, University of Gondar, Gondar, Ethiopia; 2 Department of Epidemiology and Biostatistics, Institute of Public Health, College of Medicine and Health Sciences, University of Gondar, Gondar, Ethiopia; 3 Department of Physiology, School of Medicine, College of Medicine and Health Sciences, University of Gondar, Gondar, Ethiopia; Indian Institute of Technology Kanpur, INDIA

## Abstract

**Introduction:**

Malnutrition is one of the most devastating problems in Ethiopia and is inextricably linked with poverty. Women in the reproductive age group and children are most vulnerable to malnutrition due to low dietary intakes, inequitable distribution of food within the household, improper food storage and preparation, dietary taboos, infectious diseases, and care. Therefore, this study aimed to assess the spatial distribution and determinants of undernutrition among reproductive age of Ethiopia.

**Methods:**

The study was based on the 2016 Ethiopian Demographic and Health Survey. The study included a total sampled weight of 15,139 women aged 15–49 years. The ArcGIS version 10.7 and SaTScan version 9.6 statistical software were used for exploring the spatial distribution of undernutrition. A multilevel logistic regression model was fitted to determine the individual and community level factors associated with women undernutrition. Finally, the factors which were significant at 95% confidence interval were reported.

**Result:**

The spatial analysis revealed that women undernutrition was significantly varied across the country. The SaTScan analysis identified a total of 144 significant hotspot areas of maternal undernutrition with three significant spatial windows. Of these, 134 clusters were primary. The primary spatial window was identified in the southeast Tigray, northwest Afar, central and north Amhara regions (LLR = 57.48, P<0.01, RR = 1.51). Age at first marriage (AOR = 1.57: CI 1.33, 1.99), middle wealth index (AOR = 3.15: CI 1.4, 6.97), rural residence (AOR = 2.82: CI 1.22, 6.52), being in Afar region, Tigray region and Harari region (AOR = 4.88: CI 1.71, 13.91), (AOR = 4.17: CI 1.57, 11.06) and (AOR = 3.01: CI 1.05, 8.68) respectively were significantly associated with women undernutrition.

**Conclusion:**

In Ethiopia, undernutrition had significant spatial variations across the country. Residence, age at first marriage, wealth index and region were significantly associated with undernutrition. Therefore, public health interventions that reduce reproductive age women undernutrition and enhance women awareness towards undernutrition in hotspot areas of undernutrition is crucial.

## Introduction

Undernutrition is a lack of adequate energy, protein, and micronutrients to meet basic requirements for body maintenance, growth, and development [1]. Undernutrition of women is one of the most devastating and less addressed public health problems worldwide and is inextricably linked with poverty [2,3]. It has great impact on economic productivity of adults, height of children, school achievement and risk for the birth weight of the offspring [4]. The World Health Organization(WHO) estimated that more than half millions of women’s death was resulting from undernutrition related problems[5]. Women undernutrition has great impact to their child besides to their health. A chronically undernourished woman is more likely to give birth to an undernourished child, causing the cycle of undernutrition to be repeated over generations [6,7]. In Africa, about 15% of newborns are of low birth weight and in South Asia 27% of births are of low birth weight [8]. This is due to maternal nutritional status.

A study conducted in 49 countries show that women are at high risk of low body mass index and underweight compared to man [9]. This is due to reproductive age women are most vulnerable to low dietary intakes, inequitable distribution of food within the household, improper food storage and preparation, dietary taboos, infectious diseases, and low care [10,11]. Individual and community level factors can affect the undernutrition status of women; for instance, there is a discrepancy in rural and urban residence. A systematic study done in Africa region show that rural women were 68% more likely to be undernourished compared with their urban [12–15]. Studies indicate that women with lower educational level are more likely to be undernourished than highly educated women [16,17]. The number of parity is also the important determinant factor of women undernutrition, increased number of children increases undernutrition by 38% and women who utilize unsafe water are 99% at risk of undernutrition [18,19].

The distribution of undernutrition various across the country. It is concentrated among rural residents and poor societies [20]. Literatures indicate that undernutrition varies across region and geographic areas [9,21]. For instance, it was 47.9% in Northwest Tigray, 37.2% in the Afar region [22,23]. Therefore, to design tailored public health interventions, identification of geographical areas with a high prevalence of undernutrition using geographical information system and spatial analysis is very crucial [24].

Although the magnitude of undernutrition among reproductive age group women is a common problem in Ethiopia, there are limited studies on this aspect that identify the hotspot areas of women undernutrition and the determinants of undernutrition at the national level. Therefore, this study will help health practitioners and policymakers to identify, implement, and evaluation of evidence-based interventions for the problem. It will also benefit the community by giving insight into the risk factors and preventives of undernutrition.

## Methods

### Study design, setting and period

Secondary data analysis was done based on the EDHS 2016 data. This survey was the fourth survey conducted in the country. Ethiopia is situated in the Horn of Africa, and is the 13th in the world and 2nd in Africa’s most populous country. The data were conducted in 9 regions and 2 city administrations of Ethiopia from January 18, 2016, to June 27, 2016 [25]. A community based two-stage stratified cluster sampling technique has been employed. In the first stage, 645 EAs (202 in urban areas and 443 in rural areas) were selected with probability proportional enumeration areas were selected. In the second stage, 28 households per enumeration area were selected with an equal probability systematic selection per enumeration area.

### Study variables

The outcome variable for this study was undernutrition status of non–pregnant reproductive age women which was defined as the BMI of women less than 18.5 (Yes = 1) and with BMI greater than or equal to 18.5(No = 0) [26]. In this study both individual level and community level factors were included as independent variable. The individual level variables were the women current age, marital status, educational status, occupational status, religion, age at first marriage of women, decision on earning of money, and wealth index. The residence, region, community poverty and community education level of women were the community level variables included in this study.

### Data processing and analysis

Data were weighted before doing any statistical analysis using sampling weight, primary sampling unit, and strata before any statistical analysis to restore the representativeness of the survey and to take into account the sampling design and get reliable statistical estimates. Descriptive statistics and analytical analysis were performed using STATA version 14 statistical software

### Spatial analysis

ArcGIS version 10.7 and SaTScan version 9.6 statistical software were used for exploring the spatial distribution, global spatial autocorrelation, spatial interpolation, and for identifying significant hotspot areas of women undernutrition.

### Spatial autocorrelation analysis

The spatial autocorrelation (Global Moran’s I) is the correlation coefficient for the relationship between a variable and its surrounding value, it measures the overall spatial autocorrelation of women undernutrition. Moran’s I value ranges from-1 to 1 [27]. A value close to 1 shows a strong positive spatial autocorrelation whereas a value close to -1 shows a strong negative spatial autocorrelation. If Moran’s I close to 0, it indicates that there is no spatial autocorrelation. A statistically significant Moran’s I value (p < 0.05) can lead to rejection of the null hypothesis (undernutrition is randomly distributed) and indicates the presence of spatial autocorrelation [27].

Anselin Local Moran’s I used to investigate the local level cluster locations of stillbirth in terms of positively correlated (high-high and low-low) clusters or negatively correlated (high-low and low-high). A positive value for ‘I’ indicated that a case had neighboring cases with similar values, part of a cluster. A negative value for ‘I’ indicated that a case was surrounded by cases with dissimilar values an outlier.

### Spatial interpolation

The spatial interpolation technique was used to predict women undernutrition on the un-sampled areas in Ethiopia based on sampled measurements. There are various deterministic and geostatistical interpolation methods. Among the interpolation techniques, ordinary Kriging and empirical Bayesian Kriging are the best interpolation methods since they optimize the weight. Kriging spatial interpolation method was used in this study for predicting women undernutrition in unobserved areas since it had a small mean square error and residual.

### Spatial scan statistical analysis

In the spatial scan statistical analysis, Bernoulli based model was employed to identify statistically significant spatial clusters of women undernutrition using Kuldorff’s SaTScan version 9.6 software. Women with undernutrition were taken as cases and those with not undernutrition were considered as controls to fit the Bernoulli model. The numbers of cases in each location had Bernoulli distribution and the model required data for cases, controls, and geographic coordinates.

For each potential cluster, a Likelihood Ratio (LR) test statistic and the p-value was used to determine if the number of observed underweight cases within the potential cluster was significantly higher than expected or not. The scanning window with maximum likelihood was the most likely performing cluster, and the p-value was assigned to each cluster using Monte Carlo hypothesis testing by comparing the rank of the maximum likelihood from the real data with the maximum likelihood from the random datasets. The primary and secondary clusters were identified and assigned p-values and ranked based on their likelihood ratio test, based on 999 Monte Carlo replications [28].

Regarding model comparison for multilevel binary logistic regression, four models containing variables of interest were fitted using STATA software.

***Model I* (*Empty model*)** was fitted without explanatory variables to test random variability in the intercept and to estimate the intra class correlation coefficient (ICC) and PCV.

***Model II*** examined the effects of individual level characteristics,

***Model III*** examined the effect of community level variables and

***Model IV*** (***Full model***) examined the effects of both individual and community level characteristics simultaneously.

Model comparison was done using deviance (-2 log likelihood). Accordingly, the model with the lowest Deviance was selected. Statistical significance of an association was declared at p-value <0.05 in the multi-variable analysis. Adjusted Odds Ratio (AOR) with their corresponding 95% confidence interval was determined to identify factors associated with undernutrition status among reproductive age women.

### Ethical considerations

Ethical approval letter for the use of the EDHS data set was gained from the Measure DHS website https://www.dhsprogram.com. No information obtained from the data set was disclosed to any third person.

## Results

A total of 15, 139 non- pregnant reproductive age women participated in this study. Of those, (47.8% were not educated and 36% were from the Oromia region. The majority of the women (74.29%) were married and more than 43% of the women were orthodox Christian religion followers. The majority (77.84%) of the participants were rural dwellers. Regarding to wealth index of women about 26% were richest. Almost two-third of the reproductive age women who married early (<18 years old) were undernourished (Table 1).

**Table 1:** Socio- demographic and economic characteristics of participants, EDHS 2016(weighted).

Variables	Category	Undernourished	
		Yes frequency(percent)	No frequency(percent)
Age	15–20	906(28.41)	2,322(19.43)
	21–29	985(30.90)	4,563(38.18)
	30–39	783(24.58)	3,341(27.95)
	40–49	513(16.11)	1,725(14.44)
Marital status	Not married	973(30.55)	2,859(23.92)
	Married	2,214(69.45)	9,092(76.08)
Educational status	No education	1,584(49.69)	5,696(47.66)
	Primary	1,186 (37.22)	4,123(34.50)
	Secondary	266(8.33)	1,458(12.20)
	Higher	152(4.77)	673 (5.63)
Religion	Orthodox	1,382(43.35)	5,178(43.33)
	Muslim	1,149(36.07)	3,571(29.88)
	Protestant	606(19.02)	2,933(24.54)
	Others	49(1.56)	268(2.24)
Residence	Urban	465(14.59)	2,821(23.61)
	Rural	2,722(85.41)	9,130(76.39)
Wealth index	Poorest	659(20.68)	1,881(15.74)
	Poor	604(18.94)	2,136(17.88)
	Middle	675(21.20)	2,229(18.65)
	Rich	656(20.57)	2,357(19.73)
	Richest	593(18.61)	3,348(28.01)
Region	Tigray	350(10.97)	742(6.21)
	Afar	46(1.46)	76(0.64)
	Amhara	790(24.77)	2878(24.08)
	Oromia	1248(39.17)	4238(35.46)
	Somalia	126(3.94)	308(2.58)
	Benishangul	28(0.89)	119(1.00)
	SNNP	449(14.08)	2709(22.67)
	Gambela	13(0.41)	29(0.24)
	Harari	6(0.21)	28(0.23)
	Addis Ababab	114(3.57)	759(6.36)
	Dire dawa	18(0.54)	64(0.54)
Occupation	No job	1727(54.19)	5801(48.54)
	Professional/technical	376(11.79)	2007(16.79)
	Marchant/Sale	751(23.57)	2807(23.49)
	Unskilled/manual	140(4.41)	655(5.48)
	Others	192(6.04)	682(5.71)
Age at first marriage	<18 years old	1446(65.32)	5708(62.78)
	≥18 years old	768(34.68)	3385(37.22)
Decision on earning money	Respondent alone	81(27.41)	511(30.11)
	Respondent &husband	184(62.09)	1050(61.95)
	Partner/husband alone	31(10.50)	135(7.93)
Community education	Low	2,185(68.53)	7,125(59.62)
	High	1,003(31.47)	4,826(40.38)
Community poverty	Low	1,645(51.61)	7,090(59.33)
	High	1,542(48.39)	4,861(40.67)

### Prevalence of undernutrition women in Ethiopia

The prevalence of women undernutrition in Ethiopia was 21.06% (CI: 20.4%, 21.7%). The nutritional status of women differs among residences. Of the 78% of women who lived in rural, about 85.4% were undernourished. The undernutrition status of reproductive-age women in Ethiopia was heterogeneous among educational status. Of the total women with no education, almost 50% were undernourished (Table 1).

### Spatial distribution of undernutrition among reproductive age women

**Fig 1:**
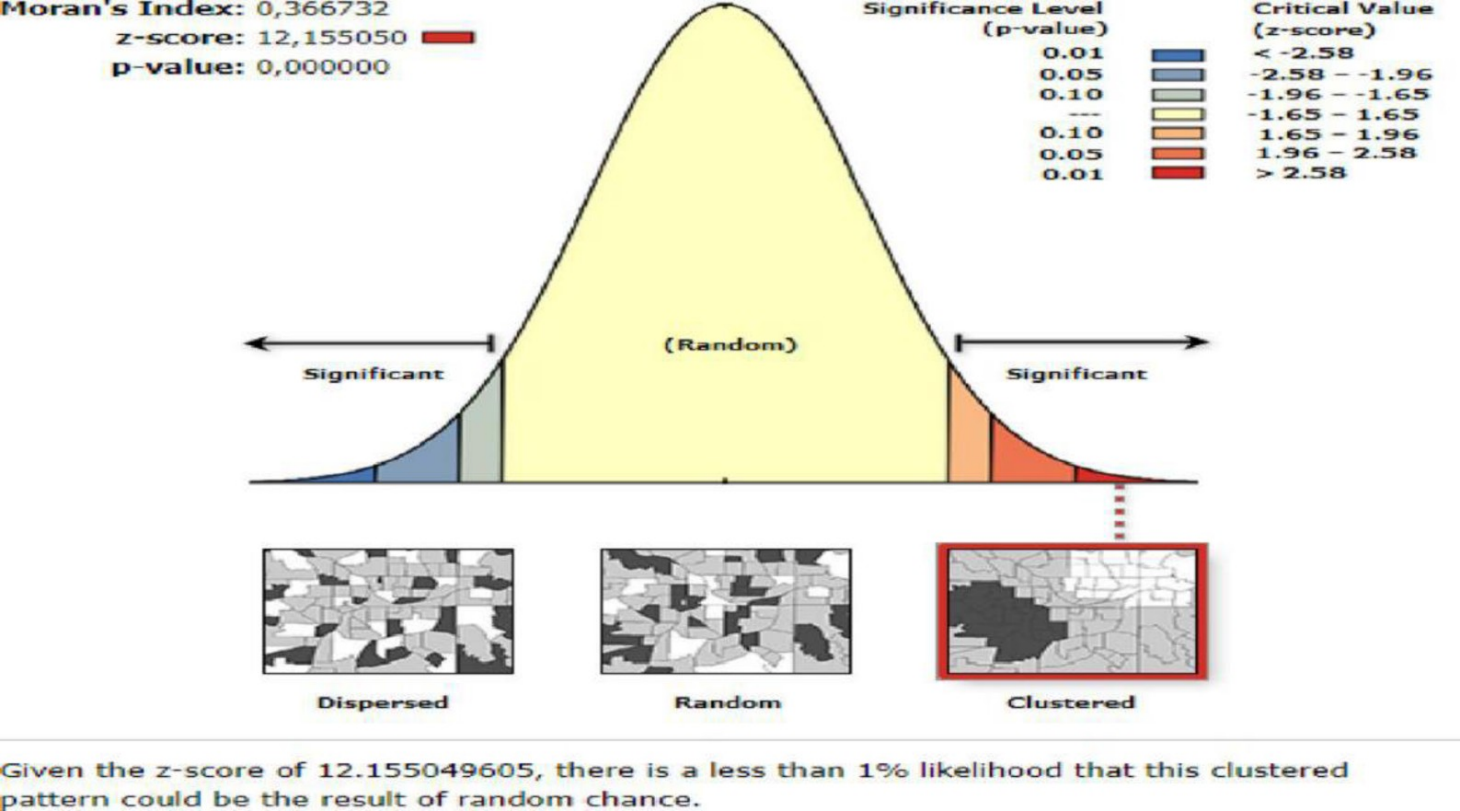
Spatial autocorrelation of undernutrition among reproductive age women in Ethiopia,2016.

**Fig 2:**
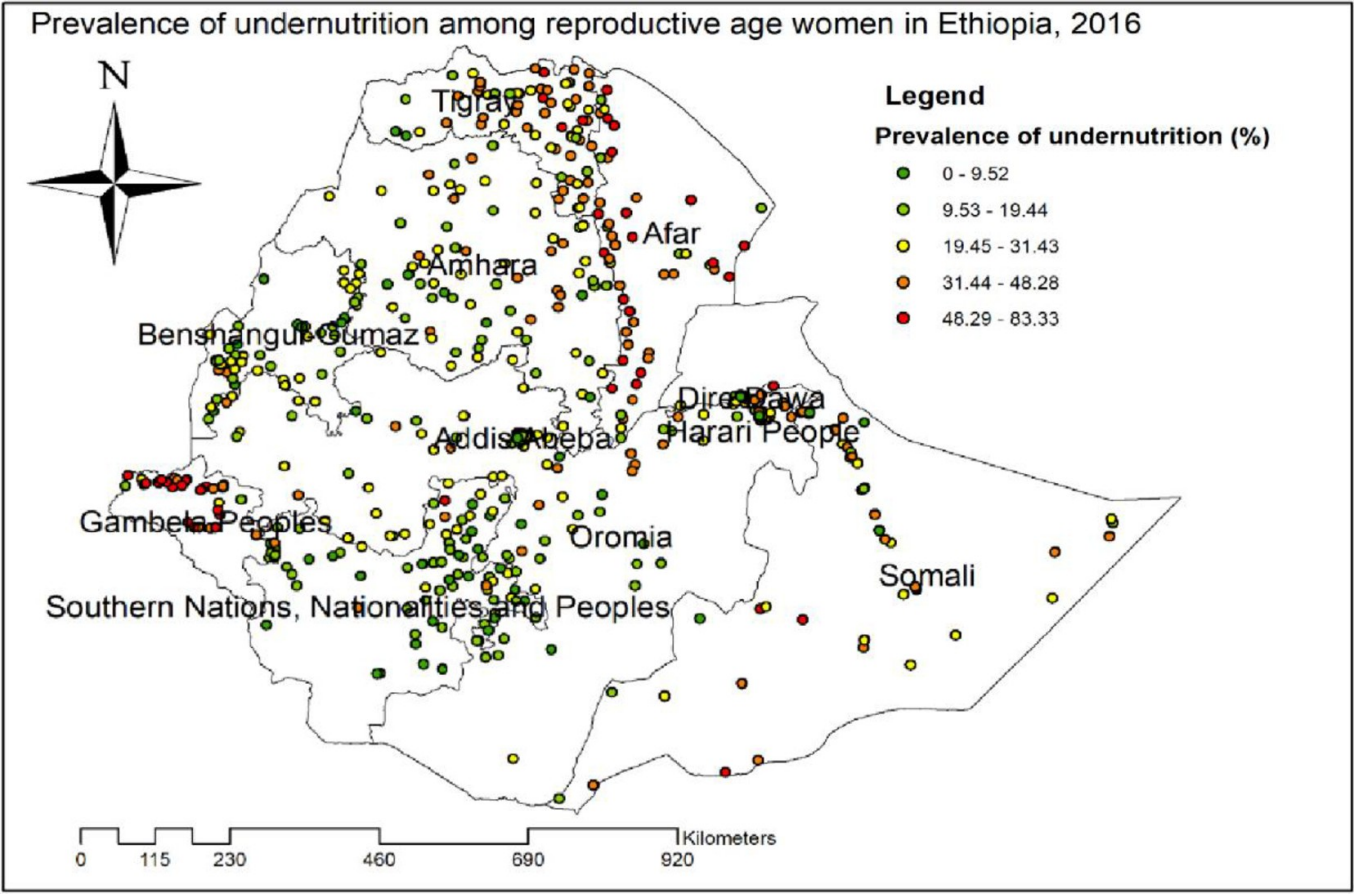
Spatial distribution of undernutrition among reproductive age women, 2016.

**Fig 3:**
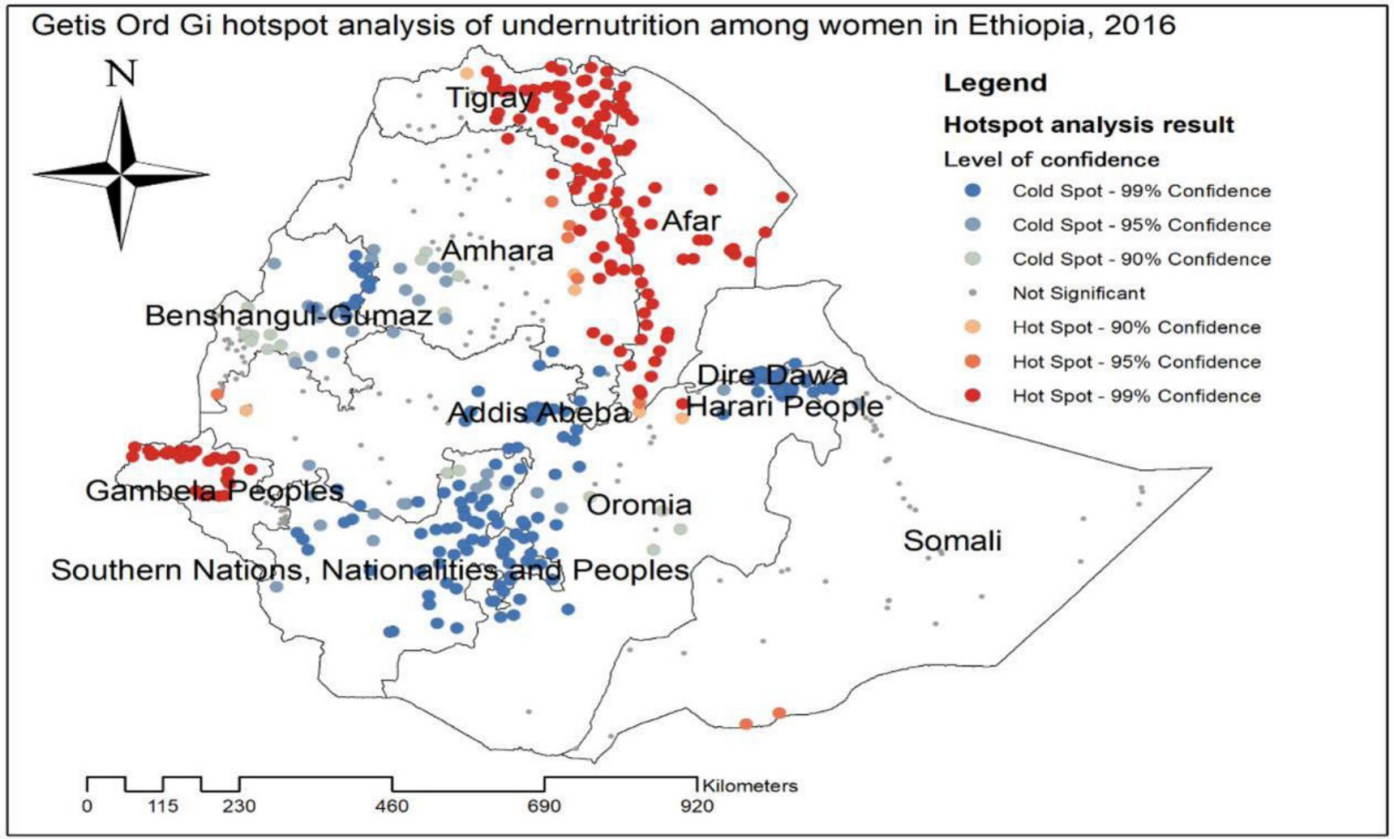
Getis Gi hotspot analysis of undernutrition among women Ethiopia, 2016.

The global spatial autocorrelation analysis of undernutrition among reproductive-age women revealed that there was a significant spatial variation of undernutrition across the country with global Moran’s I value 0.37, p-value<0.01 (Fig 1). The highest prevalence of undernutrition was identified in the entire Tigray, Gambella, and Afar regions (Fig 2). The Getis Ord Gi statistical hotspot analysis showed that the significant hotspot areas of maternal undernutrition (high prevalence of undernutrition) were located in the east Amhara, entire Afar, southwest Gambella, central and east Tigray regions while significant cold spot areas of maternal undernutrition (low prevalence of maternal undernutrition) were located in the Harari, Diredawa, Addis Ababa, northwest Oromia, northeast SNNPRs and east Gambella regions (Fig 3).

#### Spatial scan statistical analysis

**Fig 4:**
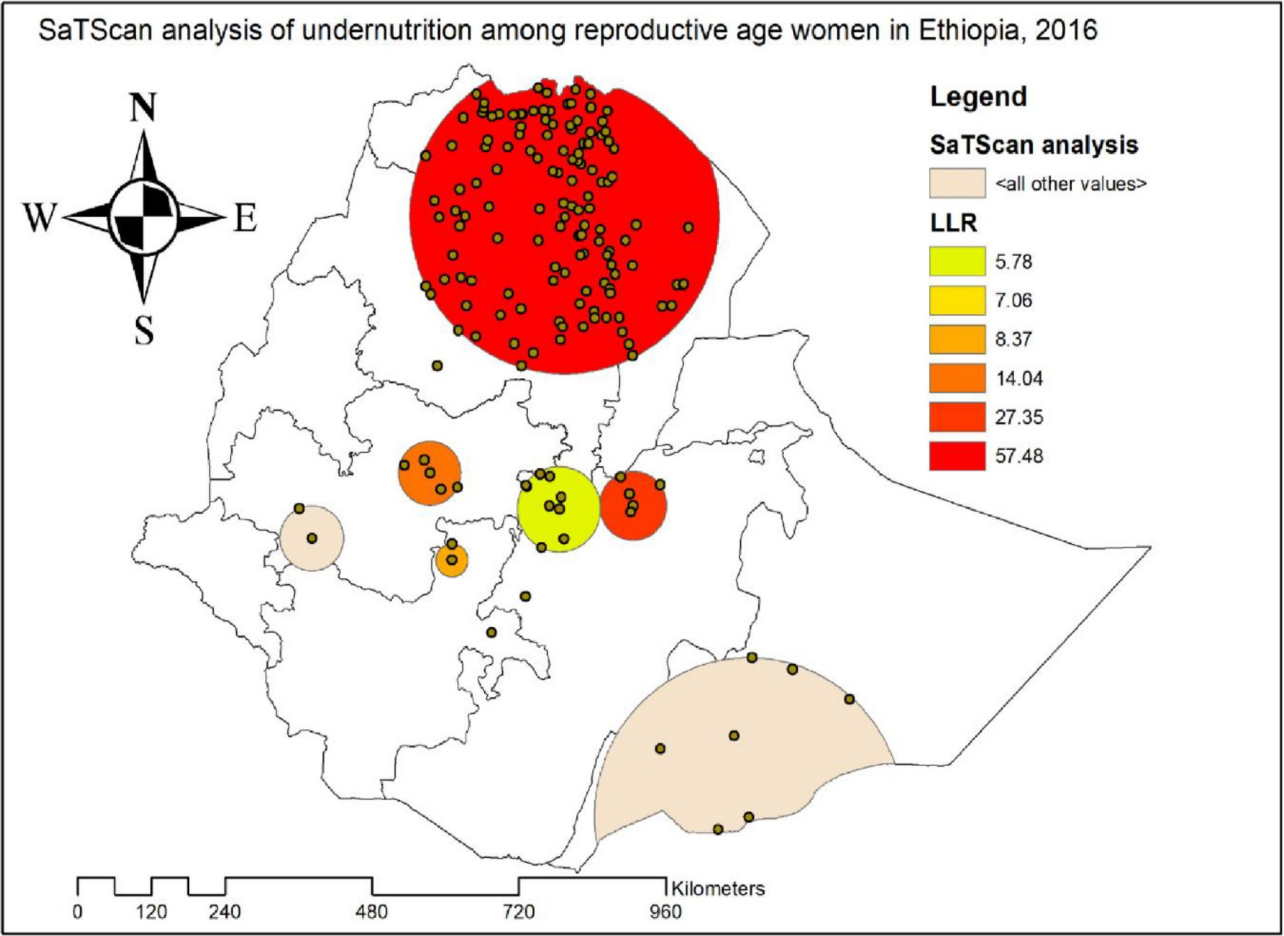
SaTScan analysis of undernutrition among women in Ethiopia, 2016.

The SaTScan analysis identified a total of 144 significant hotspot areas of maternal undernutrition with three significant spatial windows. Of these, 134 clusters were primary (most likely clusters) while 10 were secondary clusters. The primary spatial window was identified in the southeast Tigray, northwest Afar, central and north Amhara regions located at 12.677534 N, 39.348691 E with 246.36 km radius with LLR of 57.48, p-value<0.01, and RR of 1.51 (Fig 4), It showed that women within the spatial window had 1.51 times higher likelihood of undernutrition than women outside the spatial window. The secondary significant clusters were located in the central Oromia region.

#### Kriging interpolation of undernutrition

**Fig 5:**
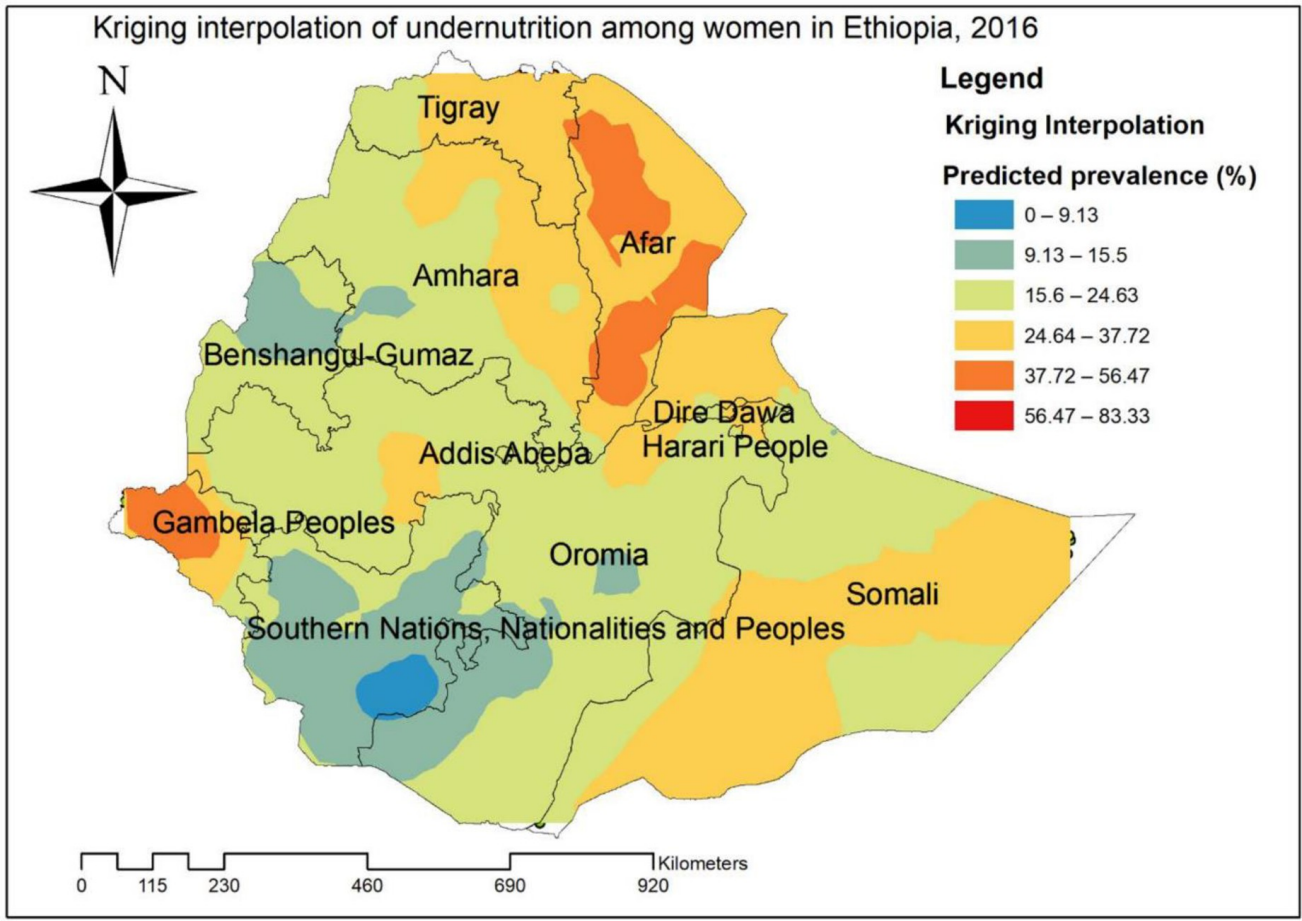
Kriging interpolation of undernutrition reproductive age women in Ethiopia, 2016.

Based on the Kriging interpolation technique; west Gambella, central and south Afar regions were predicted high risk areas of undernutrition while the predicted low risk areas of undernutrition were located in central Oromia, SNNPRs and Benishangul regions (Fig 5).

### Cluster and outlier analysis of undernutrition of reproductive age women

**Fig 6:**
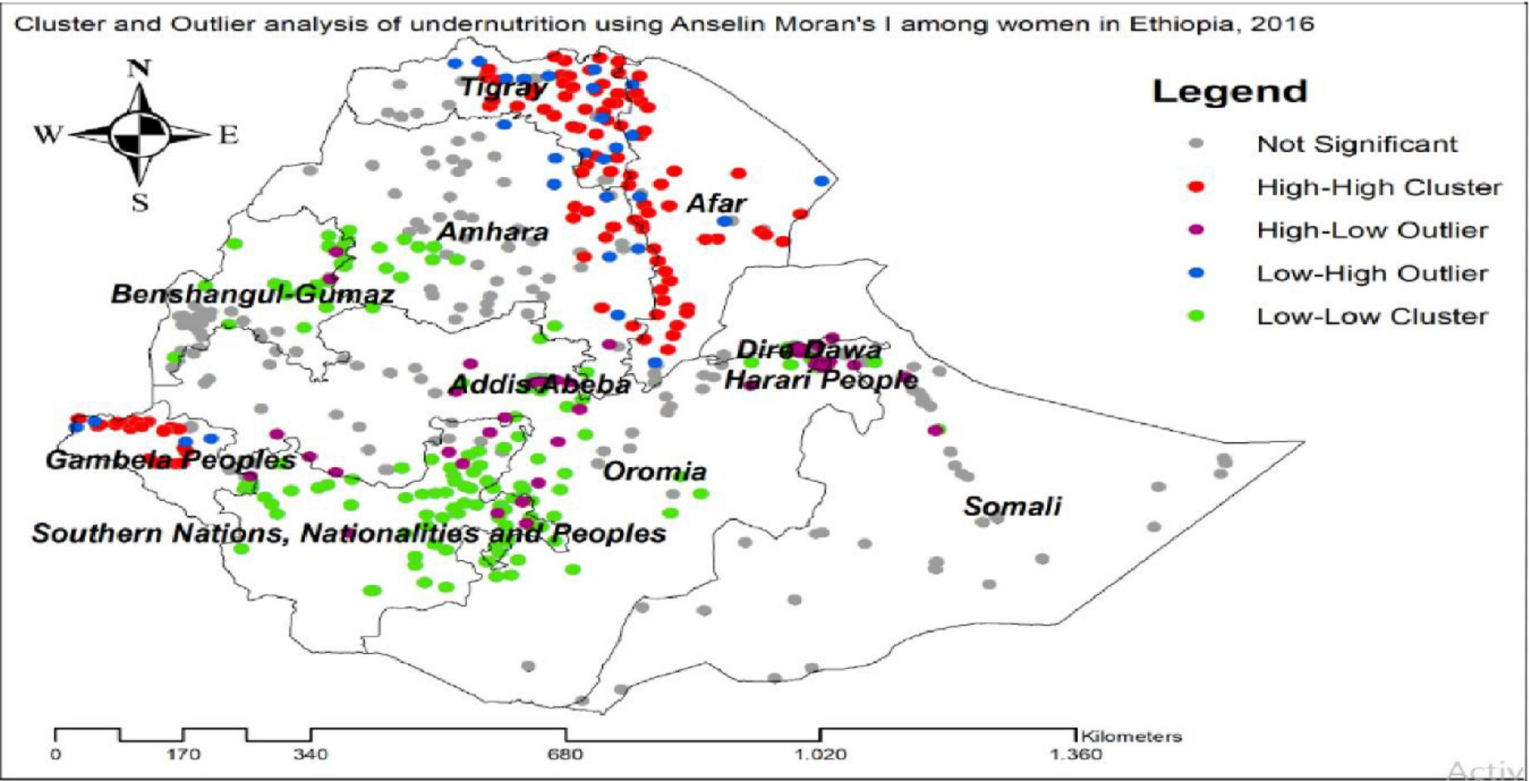
Cluster and outlier analysis of undernutrition using Anselin Moran’s I among reproductive age women in Ethiopia.

The significant cluster of women undernutrition was detected in Benishangul-Gumuz, Gambella, SNNPR, Afar, Tigray and Amhara regions. Hot spot areas for women undernutrition were found in east Tigray, east Amhara, Afar, and northwest Gambella, While the cold spot regions were found in majority areas of SNNPR, Benishangul-Gumuz, and southwest Amhara regions. Outliers were found in Addis Ababa, Dire-Dawa and Harari regions (Fig 6).

### Determinants of undernutrition status among reproductive age women of Ethiopia

#### Null multilevel logistic regression model

From the null model, variance of the random factor was 0.29 with a 95% confidence interval of (0.23, 0.37), showing heterogeneous areas. Since the variance estimate is greater than zero it indicates that there are enumeration (cluster) area differences in nutritional status among non -pregnant reproductive age women of Ethiopia, and thus multilevel analysis should be considered as an appropriate approach for further analysis.

The intra-enumeration area correlation coefficient (ICC) indicated that 8.3% with a 95% confidence interval (6.6%, 10.3%) of the total variability in nutritional status is due to differences across cluster areas. Besides, PCV revealed that 82% of the variation undernutrition was contributed to both individual and community level factors (Table 2).

**Table 2:** Community level variance of two-level mixed effect logit models predicting undernutrition of reproductive women, Ethiopia 2016.

Random effect	Null model	Full model
Community level variance	0.37	0.32
ICC (%)	15.37	8.3
PCV (%)	Reference	82
Model fitness statistics (DIC)	15583.88	1550.49

From the final model, women who were residing in the rural area increased the odds of undernutrition by 2.82 (AOR = 2.82, 95%CI 1.22, 6.52) times compared to those women residing in urban. Women who married earlier (less than 18 years old) increase the odds of undernutrition by 1.57 (AOR = 1.57, 95% CI 1.33, 1.99) times compared to those women who married at the age of 18 years old and above. The odds of women undernutrition increased by 3.15(AOR = 95% CI 1.4, 6.97) times among women with middle quantile wealth index household as compared to those women with the richest quantile wealth index. However, women from household with poorer and poorest wealth index were not associated it could be due to the presence of confounding factors. The nutritional status of women differs among the regions of Ethiopia, the odds of undernutrition of women increased by 4.17 (AOR = 95%, CI 1.57, 11.06) times in women lived in the Tigray region as compared to those women lived in Addis Ababa. Women who lived in the Afar regional state of Ethiopia increased the odds of undernutrition by 4.88 (AOR = 4.88, 95% CI 1.71, 13.91) times as compared to those women who lived in Addis Ababa. The odds of undernutrition increased by 3.01 (AOR = 95% CI 1.05, 8.68) times as compared to those women who lived in Addis Ababa (Table 3).

**Table 3:** Multilevel multivariable logistic regression of the individual and community related variables associated nutritional status of non- pregnant reproductive age women, Ethiopia, 2016.

Variable	Empty model AOR(95% CI)	Model II AOR(95% CI)	Model III AOR(95% CI)	Full model AOR(95% CI)
Age				
15–20		1.00		1.00
20–29		0.37(0.11,1.33)		0.4(0.15, 1.1)
30–39		0.33(0.09,1.17)		0.37(0.12, 1.13)
40–49		0.51(0.11, 2.33)		0.52(0.155,1.81)
Women Educational status				
No education		0.63(0.23, 1.76)		0.75(0.29,1.9)
Primary		0.82(0.32,2.07)		0.93(0.41, 2.12)
secondary		0.64(0.25,1.64)		0.67(0.27,1.61)
higher		1.00		1.00
Religion				
Orthodox Christian		1.70(0.32, 8.99)		1.21(0.35,4.10)
Muslim		2.01(0.36,11.09)		2.07(0.52, 8.14)
Protestant		1.20(0.21, 6.75)		1.52(0.42, 5.48)
Others		1.00		1.00
Residence				
Rural			1.99(1.61, 2.48)*	2.82(1.22, 6.52)*
Urban			1.00	1.00
Who decide on earning money				
Respondent alone		0.83(0.33,2.09)		0.90(0.56, 1.47)
Husband alone		0.72(0.31,1.60)		1.30(0.60, 2.81)
Respondent and husband/partner		1.00		1.00
Age at first marriage				
<18 years old		0.65(0.38, 1.12)		1.57(1.33, 1.99)*
> = 18 years old		1.00		1.00
Wealth index				
poorest		2.66(1.14, 6.23)*		1.31(0.61,2.83)
Poorer		3.35(1.53,7.31)*		1.87(0.84, 4.17)
Middle		6.32(2.86,13.97)*		3.15(1.4, 6.97)*
Richer		3.57(1.54, 8.31)*		1.78(0.79, 4.01)
Richest		1.00		1.00
Region				
Tigray			1.92(1.48,2.49)*	4.17(1.57,11.06)*
Afar			4.88(1.72,13.91)*	4.88(1.71,13.91)*
Amhara			1.70(0.58,5.21)	1.71(0.55, 5.21)
Oromia			0.74(0.28,1.92)	0.74(0.28,1.92)
Somali			0.97(029,3.25)	0.97(0.29,3.25)
Benishangul			1.14(0.31, 4.16)	1.14(0.34,4.16)
SNNP			0.52(0.17, 1.56)	0.52(0.17,1.56)
Hareri			3.01(1.04, 8.68)*	3.01(1.05, 8.68)*
Gambela			0.77(0.25,2.33)	0.77(0.26, 2.33)
Diredawa			1.15(0.43,3.05)	1.15(.4383567 3.05(0.44, 3.05)
Addis ababa		1.00	1.00	1.00
Community level women education				
Low			1.00	1.00
High			1.06(0.88,1.27)	1.15(0.64, 2.05)
Community level poverty				
Low			1.00	1.00
High			1.13(0.98,1.31)	1.00(0.55,1.84)

## Discussion

This study was conducted on the undernutrition status of non -pregnant reproductive-aged women in Ethiopia. Spatial analysis revealed that spatial distribution of undernutrition among reproductive-age women significantly varied across the country. Accordingly, from the individual-level factors, age at first marriage and wealth index were significantly associated with undernutrition of women while residence and region of women were the community-level factors which significantly associated with undernutrition of women in Ethiopia.

The spatial analysis shows that undernutrition of reproductive age women significantly varied across the country. The significant areas of hot spot areas were indicated in southeast Tigray, northwest Afar, central and north Amhara, and central Oromia region. The possible explanation could be related with economical variation, drought, food insecurity and variation in cultivation [29]. This finding suggests that public health planners and programmers should design effective public health interventions to reduce women undernutrition in these significant hotspot areas where undernutrition is high.

The odds of women undernutrition was higher among women who married at earlier(less than 18 years old) as compared with women who married at the age of 18 and above. This finding is consistent with the study done in Nigeria and Tigray [23,30]. Mothers who married earlier are less likely to utilize family planning service and other maternal and health care services so that they exposed to early and multiple child bearing, unwanted and unplanned pregnancy thus further contribute to her nutritional status [31]. Furthermore, women are responsible for their child, spouse and the whole family so there might be inequitable distribution of food within the household because they give priority for their children and husbands so that they will be vulnerable to undernutrition.

This study revealed that women with middle wealth index were more likely to be undernourished than those with richest wealth index. However, women with the poorest and poorer wealth index were not associated with undernutrition. This could be due to confounding effect. This is supported with studies conducted in Tanzania and India, undernutrition status decrease as wealth index increase [13,32]. Women who are poor are unable to purchase foods, water, clothes and good shelter. Thus they are at risk of poor hygiene and sanitation, contracting different communicable diseases and nutritional deficiencies, it could be micro nutrient deficiency or micronutrient deficiency [33].

The odds of women undernutrition was higher among women who lived in rural than their counterpart. Studies conducted in Tanzania, Ghana and Bangladesh reported similar associations [15,34,35]. Most of the rural women in Ethiopian are illiterates, they don’t have adequate information regarding balanced diet. Besides, in developing countries like Ethiopia, the bacterial and parasitic diseases have a great contribution to rural women’s undernutrition status [18]. Therefore, information education and communication and behavioral change communication regarding balanced diet, and over all nutrition is needed for rural women.

This study revealed that the women undernutrition significantly differ among regions. Women who were from the Afar region, Tigray region and Harari region were more likely to be undernourished than those women who lived in Addis Ababa. The possible explanation for this finding might be, for instance, most the Afar region women are agro-pastoralist with large family size (mean family size is 6.3). Furthermore, most of the Afar households are with food insecurity, thus they are at risk of being undernourished [22]. Regarding the Tigray region women, it is one of the region grouped under food insecure regional states with high levels of vulnerability caused by repeated shocks allied with diminished entitlements, low natural resource endowments and limited access to infrastructure. So the women are more likely to be risk of undernourished due to food insecurity [12]. Therefore, giving special attention for these regions while designing nutritional interventions is required.

### Limitation of the study

This study had some limitations. The GPS data (Latitude and Longitude) taken at enumeration area were displaced to 5 Km in urban areas and 10 Km in Rural areas for the privacy issue, this could bias our spatial result. Furthermore, due to the cross-sectional nature of the data, the temporal relationship can’t be established.

## Conclusion

In Ethiopia, undernutrition had significant spatial variations across the country. Residence, age at first marriage, wealth index and region were significantly associated with undernutrition. Therefore, improving the socioeconomic status of women and reducing the early marriage culture of girls is needed to minimize women’s undernutrition. Health professionals shall better to deliver nutritional education for rural reproductive age women. And the Ethiopian Federal Ministry of Health (FMOH) should design tailored nutritional intervention for reproductive age women who are living in the Afar, Tigray, and Harari regions.

## Abbreviation/Acronyms

AOR: Adjusted odds ratio
BMI: Body mass index
CI: Confidence interval
EA: Enumeration area
EDHS: Ethiopian demographic health survey
ICC: Intra class correlation
MUAC: Middle upper arm circumference
PCV: Power of change variance
